# The legislative framework for salt iodization in Asia and the Pacific and its impact on programme implementation

**DOI:** 10.1017/S1368980017001689

**Published:** 2017-09-07

**Authors:** Karen Codling, Christiane Rudert, France Bégin, Juan Pablo Peña-Rosas

**Affiliations:** 1 Iodine Global Network, Ottawa, Canada; 2 UNICEF Regional Office for East Asia and the Pacific, PO Box 2-154, Bangkok 10200, Thailand; 3 Nutrition Section, UNICEF Headquarters, New York, NY, USA; 4 Evidence and Programme Guidance, Department of Nutrition for Health and Development, World Health Organization, Geneva, Switzerland

**Keywords:** Salt iodization legislation, Universal salt iodization, Iodine deficiency elimination, Asia

## Abstract

**Objective:**

Fortification of food-grade (edible) salt with iodine is recommended as a safe, cost-effective and sustainable strategy for the prevention of iodine-deficiency disorders. The present paper examines the legislative framework for salt iodization in Asian countries.

**Design:**

We reviewed salt iodization legislation in thirty-six countries in Asia and the Pacific. We obtained copies of existing and draft legislation for salt iodization from UNICEF country offices and the WHO’s Global Database of Implementation of Nutrition Actions. We compiled legislation details by country and report on commonalities and gaps using a standardized form. The association between type of legislation and availability of iodized salt in households was assessed.

**Results:**

We identified twenty-one countries with existing salt iodization legislation, of which eighteen were mandatory. A further nine countries have draft legislation. The majority of countries with draft and existing legislation used a mandatory standard or technical regulation for iodized salt under their Food Act/Law. The remainder have developed a ‘stand-alone’ Law/Act. Available national surveys indicate that the proportion of households consuming adequately iodized salt was lowest in countries with no, draft or voluntary legislation, and highest in those where the legislation was based on mandatory regulations under Food Acts/Laws.

**Conclusions:**

Legislation for salt iodization, particularly mandatory legislation under the national food law, facilitates universal salt iodization. However, additional important factors for implementation of salt iodization and maintenance of achievements include the salt industry’s structure and capacity to adequately fortify, and official commitment and capacity to enforce national legislation.

In the mid-1990s, it was estimated that the inhabitants of 130 countries were at risk of iodine-deficiency disorders (IDD)^(^ ^1^ ^)^. This number has been reducing steadily over the years, to only nineteen in 2016^(^ ^2^ ^)^. This significant reduction in iodine deficiency has been achieved through universal salt iodization (USI), which is recommended as a safe and sustainable strategy to eliminate IDD^(^ ^3^ ^)^. The proportion of households consuming iodized salt globally has increased from less than 20 % in the early 1990s^(^ ^4^ ^)^ to 75 % today^(^ ^5^ ^)^. Salt iodization has been recognized as a global public health success story^(^ ^1^ ^)^ and one of the most cost-effective nutrition interventions, with a benefit:cost ratio of the order of 30:1^(^ ^6^ ^)^. The WHO recently published a comprehensive systematic review of the effects of salt iodization^(^ ^7^ ^)^ and guidelines on salt iodization, recommending that all food-grade (edible) salt, which is used in households and for food processing, be fortified with iodine for the prevention and control of IDD^(^ ^8^ ^)^. There is global consensus that strategies for salt reduction and USI are compatible^(^ ^9^ ^)^.

The importance of legislation for salt iodization, in particular mandatory legislation, has been recognized^(^ ^10^ ^,^ ^11^ ^)^. A database maintained by the Iodine Global Network (IGN) indicates that 130 out of 197 countries have mandatory legislation for the iodization of at least household/table salt or salt for food processing^(^ ^2^ ^)^. In 2008, UNICEF estimated that globally fifty-five out of 117 low- and middle-income countries had some form of legislation^(^ ^12^ ^)^. Countries with supportive legislation have shown a greater improvement in household coverage of iodized salt in the last decade: from 49 to 72 % compared with 40 to 49 % in those without legislation^(^ ^10^ ^)^. The present paper reports on findings of a review undertaken among Asian and Pacific Island countries* on their salt iodization legislation and its impact on USI programme achievements.

## Methods

We collected and reviewed draft and existing legislation, relevant programme documentation and policy papers on salt iodization from thirty-six countries in Asia. English translations of the abovementioned documents were obtained from UNICEF country offices in the countries listed in Table 1 in 2012 and updates were requested in 2015/16. An additional search was conducted of the WHO-hosted Global Database of Implementation of Nutrition Actions (GINA) and through WHO regional offices. Key parameters were extracted for analysis from the documents: name, year and nature of legislation, mandatory or voluntary, objective, scope, and whether the salt iodization standard is in the legislation or a separate regulation. Implementation details given in the legislation or supporting documents, such as coordination and enforcement authority, labelling/packaging requirements, internal and external regulatory monitoring requirements, and penalties, were also reviewed. If the country legislation specifies salt for human and animal use, and does not mention exclusion of salt for food processing, it was assumed that the country had USI. Basic information on the current status of the countries’ salt iodization programmes and household consumption of iodized salt data was sought from global WHO, UNICEF and IGN databases and country programme documents.

**Table 1:** Basic information on iodized salt legislation in the thirty-six countries reviewed

Country	Existence of legislation	Legislation name	Year of current legislation	Nature of legislation	Mandatory or voluntary	Scope	Salt iodization standard	Salt iodization level (mg/kg)	Notes/comments
1. Afghanistan	Existing	Regulations on Iodizing Salt	2011	Regulation under Health Law	Mandatory	USI	In Regulation	P: 30–50 R: 30–50 H: 15	
2. Bangladesh	Existing	1. Protection Law of Diseases Caused by Lack of Iodine 1989 (LAW) 2. Diseases for Deficiency of Iodine Prevention Act 1994 (REGULATION) 3. Salt Policy endorsed by Cabinet Decision, 25 August 2011	1989	Stand-alone Law and Act	Mandatory	Food processing salt excluded	In Law	P: 40–50 R: ≥20	New legislation is reportedly under development
3. Bhutan	None						In Operational Guidelines	P: 50 R: 25 H: 15	Although there is no legislation, non-iodized salt has been banned by the National Assembly and the sole salt plant is state-owned
4. Brunei Darussalam	Existing	Public Health (Food) Regulations	2000	Regulation under Food Act	Voluntary		In Regulations	20–40	
5. Cambodia	Existing	1. Sub-Decree on Management of Iodized Salt Exploitation, 20 October 2003 2. Prakas on the Procedure of Management of Exploitation of Iodized Salt, 24 February 2004 3. Joint Prakas on the Management Procedures of All Kinds of Iodized Salt Exploitation, 2 July 2004	2003	Stand-alone Sub-Decree	Mandatory	USI; food processing specifically included	In Sub-Decree and Prakas	P: 50–60R: 30–60	
6. China	Existing	Regulation on Edible Salt Iodization as a Means to Eliminate IDD	1994	Stand-alone State Council regulation	Mandatory	USI; food processing specifically included	Separate standard: National Standard for Iodized Salt, GB 26 878–2011	25–30±30 %	
7. Cook Islands	None								
8. Democratic People’s Republic of Korea	None						Separate standard: National Standard for Salt, KPS 320:2014	50±5	The State Planning Commission has a plan to add mandatory requirements for salt iodization to an existing Salt Law
9. Fiji	Existing	Standards on Salt and Spices, Food Safety Regulations	2009	Regulation under Food Act	Mandatory	USI	In Regulations	20–30	
10. India	Existing	Food Safety and Standards Act 2006 – Prohibition and Restrictions on Sales Regulations 2011 and Food Products Standards and Food Additives Regulations 2011	2011	Regulation under Food Act	Mandatory	Household only; food processing and livestock salt specifically excluded	In Regulations	P: >30 R: >15	
11. Indonesia	Existing	Presidential Decree No. 69 1994 on Supply of Iodized Salt	1994	Stand-alone Presidential Decree	Mandatory	Household and livestock; food processing appears to be excluded in some supporting regulations	Separate standard: National Standards Office SNI 2556–2010 on Iodized Consumption Salt	>18	Indonesia has a complex series of legislation for salt iodization some of which is ambiguous or contradictory
12. Kiribati	Existing	Food Regulations and Standards under Food Safety Act 2006	2014	Regulation under Food Act	Mandatory	Household and food processing	In Regulations	20–30	Kiribati standard follows regional standard developed in 2007
13. Lao People’s Democratic Republic	Existing	Prime Minister’s Legislation on Salt Iodization No. 42/PM	1991	Stand-alone Law	Mandatory	USI	Separate standard: Ministry of Health Standard for Iodized Salt No. 102/MH 2005	P: 40–60 R/H: >20	
14. Malaysia	Existing & planned	Ministers Direction under Regulation 285 of the Food Regulations 1985 under the Food Act 1983	1999 (Sabah) 2008 (Sarawak)	Regulation under Food Act	Mandatory	Household and food processing; livestock salt excluded	In Ministers Direction	20–30	Salt iodization is currently required in only 2 of Malaysia’s 16 states
15. Marshall Islands	Draft	DRAFT Food Safety Regulations include standards for iodized salt		Regulation under Food Act					
16. Maldives	None								
17. Micronesia (Federated States of)	Draft	DRAFT Food Control Regulations include standards for iodized salt		Regulation under Food Act					
18. Mongolia	Existing	Law of Mongolia – Salt Iodization and Prevention of Iodine Deficiency	2003	Stand-alone Law	Mandatory	Household and food processing (specifically included); livestock salt excluded	Separate standard: Technical Requirements for Edible Iodized Salt MNS 5046: 2001	30±5	
19. Myanmar	Existing & planned	Ministry of Mines Notification No. 40/98	1998	Notification under Salt Enterprise Law	Mandatory	USI	Separate standard: Ministerial Directive of Ministry of Mines	P: 40–60 W: 30 R: >15 H: 15	
20. Nauru	Draft	DRAFT Food Safety Regulations include iodized salt standards		Regulation under Food Act					
21. Nepal	Existing	Edible Iodized Salt (Production and Distribution) Act 2052	1996	Stand-alone Act	Mandatory	USI	Separate standard: Nepal Food Standard 5 February 2001 under Food Act B.S. 2012 (AD 1966/67)	P: 50 R: 30 H: 15	
22. Niue	Draft	DRAFT Food Safety Regulations include standard for iodized salt		Regulation under Food Act					
23. Palau	None								
24. Pakistan	Draft	DRAFT The IDD Control Bill	2009	Stand-alone Bill	Mandatory	USI; food processing specifically included	Separate standard: Standard Specification for Iodized Food Grade Salt, 2nd revision, 2008, of Pakistan Standard and Quality Control Agency	P: 30 R: 15–25	National legislation was drafted in 2009 but has yet to be passed. Some administrative units of Pakistan have their own legislation
25. Papua New Guinea	Existing	Amendment of the Pure Food Standards under the Pure Food Act chapter 233	1995	Regulation under Food Act	Mandatory	USI	In Standards	Salt: 30–50 Table salt: 40–70	
26. Philippines	Existing	1. Republic Act No. 8172 ‘An Act Promoting Salt Iodization Nationwide and for Related Purposes’ 2. Department of Health Revised Implementing Rules and Regulations of Republic Act 8172, ‘An Act Promoting Salt Iodization Nationwide and for Related Purposes’, 2004 3. Food and Drug Administration (FDA) Memorandum Circular: ‘Guidelines for salt manufacturers, importers, wholesalers, repackers and distributors to ensure adequate iodization of iodized salt, and for other purposes’	1995	Stand-alone Act	Mandatory	USI; food processing specifically included	Separate standard: FDA Circular 2013–007. Amendment of Bureau Circular No. 2007–009 on the Standard Iodine Level of Salts for Strict Compliance of Iodized Salt Manufacturers or Processors	30–70	
27. Samoa	Draft	DRAFT Food (Safety and Quality) Regulations 2014 include standards for iodized salt		Regulation under Food Act	Mandatory (draft)			20–30	Samoa standard follows regional standard developed in 2007
28. Singapore	Existing	Food Regulations	1998	Regulation under Food Act	Voluntary		In Regulations	25–40	
29. Solomon Islands	Existing	Standards on Salt and Spices under Pure Food (Food Control) Regulations 2010	2010	Regulation under Food Act	Mandatory	USI	In Regulations	20–30	
30. Sri Lanka	Existing	Regulation under Section 32 of the Food Act, No. 26 of 1980 – Food (Iodization of Salt) Regulations	2005	Regulation under Food Act	Mandatory	Household and food processing (specifically included); livestock salt excluded	In Regulations	15–30	
31. Thailand	Existing	Public Health Ministry Announcements on Edible Salt (2011); Fish Sauce (2010); Seasoning from Fermented Soya Bean (2010); Seasoning Saline (2010)	2010 & 2011	Announcements under Food Act	Mandatory	USI; food processing specifically included	In Announcements	20–40	Announcements require iodization of all edible salt, including for food processing, except for fish sauce, soya sauce and salt brine which can be produced with iodized salt or iodine directly
32. Timor Leste	Draft	DRAFT Decree-Law No.___/2010 of June 30, 2010 Iodization of Salt Law	2010	Stand-alone Law	Mandatory	USI – food processing specifically included		20–50	Draft from 2010 has yet to be passed
33. Tonga	None								
34. Tuvalu	Draft	Draft Food Safety Regulations include standards for iodized salt		Regulation under Food Act					
35. Vanuatu	Draft	Draft Food (Control) Regulations 2014 include standards for iodized salt		Regulation under Food Act	Mandatory (draft)			20–30	Vanuatu standard follows regional standard developed in 2007
36. Vietnam	Existing (and old)	Existing: Decree 09 – Regulation on Micronutrient Fortification of Foods (2016) Old: Government Decree on Production and Supply of Iodized Salt for Human Consumption, No. 163	2016 2005	Stand-alone Decree (old and new)	New decree is mandatory; old decree is voluntary	New decree: household and food processing; livestock is not mentioned	Separate standard: National Technical Regulation on Iodized Salt (QCVN 9-1:2011/BYT)	20–40	New Decree makes salt iodization mandatory, unlike old Decree, and includes the mandatory fortification of wheat flour and cooking oil. It came into force on 15 March 2017. Standards are under review

IDD, iodine-deficiency disorders; USI, universal salt iodization; P, production; R, retail; H, household; W, warehouse.

## Results

Key information on the status of salt iodization legislation in the reviewed countries is shown in Table 1.

### Existence of legislation

Out of the thirty-six countries reviewed, twenty-one had salt iodization legislation, eighteen mandatory. Legislation was voluntary in Brunei and Singapore where a standard for iodized salt is available but it does not apply to all salt. Legislation was also voluntary in Vietnam at the time the review started, although Vietnam passed new legislation on 28 January 2016 that reinstated mandatory salt iodization, effective as of March 2017^(^ ^13^ ^)^.

### Draft legislation

Nine countries currently have draft legislation. These include seven Pacific Island countries that have draft Food (Safety/Control) Standards which include iodine requirements in salt.† Pakistan has draft national legislation dated 2009 that is awaiting endorsement. The endorsement process has stalled since the power of the Ministry of Health was devolved to the provinces in 2011. In the absence of national legislation, several states and territories have passed their own legislation.‡ Timor Leste has draft national legislation dated 2010; limited efforts have taken place since it was drafted to enact it.

Bangladesh, Lao People’s Democratic Republic (Lao PDR), Malaysia and Myanmar are revising or updating their legislation. However, drafts of the new legislation were not made available. It should be noted that while the existence of or plans for revised legislation from several countries was noted in 2012, the situation remained unchanged when the review was updated in 2015/16. In Malaysia, however, the Ministry of Health has advised that nationwide mandatory salt iodization legislation is in the final stages of government approval.§

### Countries without legislation

Six of the thirty-six countries had no existing or draft legislation (Table 1). Although Bhutan does not currently have official legislation for salt iodization, in 1984, the National Assembly (Parliament) banned the importation of non-iodized salt and commissioned the establishment of a salt iodization plant as a joint venture between a private firm and the government (Bhutan Salt Enterprise). All salt is imported into Bhutan already packed and iodized, primarily from India. Inadequately iodized salt is iodized by Bhutan Salt Enterprise.‖ In the Democratic People’s Republic of Korea (DPRK), there is no legislation for salt iodization although the government has a policy to produce iodized salt and the State Planning Committee has a plan to amend an existing Salt Law to include requirements to produce sufficient iodized salt to meet national needs.¶ The Maldives does not currently have legislation. All salt is imported and the planned focus is on strengthening the quality assurance of imported salt.**

### When legislation was passed

Bangladesh was the first country in Asia to pass national legislation in 1989.†† Most countries passed their legislation in the 1990s, following the 1990 World Summit for Children which established the goal of virtual elimination of IDD^(^ ^14^ ^)^ and the 1994 recommendation of WHO and UNICEF for USI^(^ ^3^ ^)^. Cambodia, Mongolia, Afghanistan and the Solomon Islands passed legislation as late as the 2000s and Kiribati only in 2016. On the other hand, Afghanistan, India, Thailand and Vietnam have already updated their initial legislation; Vietnam issued its third salt iodization legislation at the start of 2016.

### Nature of legislation

The thirty countries with draft and existing legislation have utilized different mechanisms to legislate for iodization of salt. The majority (seventeen) have developed mandatory standards or regulations for iodized salt under their Food (Safety) Act/Law. Most of these countries simply amended their salt standard/technical regulation for ‘food-grade salt’ or ‘edible salt’ to specify the iodine content, in addition to other existing criteria such as purity or moisture. For example, in Papua New Guinea, the legislation provides the following specifications for salt:

(a) ‘is sodium chloride, free from dirt; and
(b) shall contain, on a water-free basis and –

I. not more than 1 % of sulphates; and
II. not more than 0·1 % of matter insoluble …
III. not more, in total of 0·5 % calcium and magnesium chlorides; and

(c) shall be iodized.’

Three of the seventeen countries with salt iodization legislation under their Food (Safety) Act/Law have enacted more detailed regulations than a mandatory standard for iodized salt, such as Sri Lanka’s ‘Iodization of Salt Regulation’ and India’s ‘Prohibition and Restrictions on Sales Regulations’ that place restrictions on the sale of common salt unless it is iodized. Malaysia has also placed restrictions on sale of non-iodized salt in two states in its Food Act.

Two countries have developed a regulation under an alternative Act/Law; Afghanistan issued a regulation on iodizing salt under its Health Law and Myanmar issued a notification under its Salt Enterprise Law.

The remaining eleven countries with existing or draft legislation for salt iodization have issued a separate, stand-alone law or equivalent. For example, Indonesia issued a Presidential Decree on ‘Supply of Iodized Salt’, Lao PDR issued the ‘Prime Minister’s Legislation on Salt Iodization’, the Philippines issued ‘An Act Promoting Salt Iodization Nationwide and for Related Purposes’ and Mongolia issued the ‘Law of Mongolia – Salt Iodization and Prevention of Iodine Deficiency’. Such legislation usually has a number of supportive regulations, guidelines or implementing rules that detail how the legislation should be implemented.

### Objective of legislation

Although all salt iodization legislation requires the iodization of salt, existing legislation has three different objectives: (i) non-iodized salt is banned or only iodized salt is allowed; (ii) all salt within the scope of the legislation should be iodized as per national standards; and (iii) guides the production of iodized salt but allows non-iodized salt to be produced. The different objectives guide how the legislation is enforced. Enforcement of legislation with the first objective is focused on identifying and removing non-iodized salt whereas under the second objective, the focus is on ensuring all salt is iodized as per national standards. In many countries where the legislation refers to the iodization of all salt, the focus of enforcement is on controlling the registration of salt producers and ensuring production of quality iodized salt, rather than just ensuring that all salt, regardless of who makes it or its quality, is iodized. In the third category, the legislation guides the iodization of salt, for example who can produce it, but does not emphasize that all salt within the scope of the legislation must be iodized. Of the thirty countries with existing and draft legislation, twenty ban non-iodized salt or allow only iodized salt (first objective), six require all salt to be iodized (second objective) and four guide iodized salt production but do not ban non-iodized salt (third objective). The latter category includes the three countries with voluntary legislation.

### Scope of legislation

USI refers to ‘the iodization of all human and livestock salt, including salt used in the food industry’^(^ ^18^ ^)^. Recent, updated WHO guidelines for the fortification of food-grade salt with iodine emphasize the importance of fortifying salt in food processing^(^ ^8^ ^)^.

A total of thirteen of twenty-one countries with existing legislation have universal salt iodization by the above definition. Of the thirteen countries with universal scope, six specifically mention the inclusion of the salt for food processing. Remaining countries (eight) exclude either salt for food processing or for animal consumption from existing legislation. Bangladesh specifically excludes salt for food processing and legislation for Indonesia is ambiguous; some documents include it while others do not. Salt for livestock is specifically excluded from the scope of the legislation in Malaysia, Mongolia and Sri Lanka. Indian legislation excludes salt for both food processing and livestock.

The iodization of all salt for human and animal consumption, including salt for food processing, is recommended in order to ensure that all salt is iodized and that iodine intakes are adequate^(^ ^8^ ^,^ ^15^ ^)^. An increasing amount of salt is consumed as salt in processed foods^(^ ^16^ ^)^. If this salt is not iodized, it is possible that iodine consumption will be insufficient to achieve adequate iodine intake levels. Animal salt should be iodized because the health and productivity of animals is enhanced by adequate iodine intakes^(^ ^17^ ^)^ and animal products, such as milk, can be an important source of iodine^(^ ^18^ ^)^. It is also important to iodize all these types of salt to avoid the availability of non-iodized salt for food processing and animal consumption, which can then leak into the market for household salt.

### Salt iodization standards

In 2014, WHO issued updated recommendations for the amount of iodine to add to salt^(^ ^8^ ^)^. The recommended iodine levels depend on the total amount of salt consumed as table salt and from processed foods. The guidelines assume 30 % losses of iodine from production to household but note that losses could vary widely depending on the iodization process, the quality of the salt and packaging materials, and the climatic conditions. The guidelines also assume a 92 % iodine bioavailability. Taking these assumptions into account, the amounts of iodine recommended are intended to provide the recommended nutrient intake of 150 µg iodine/d. The guidelines further note that salt iodization and salt reduction are compatible, hence they recommend addition of higher levels of iodine for lower intakes of salt and that urinary iodine concentrations should be monitored to guide adjustments of iodine concentrations in salt.

If estimated salt intake is 5 g/d or lower, as recommended to reduce the risks of non-communicable diseases^(^ ^19^ ^)^, the recommended iodine level is on average 39 mg/kg ±10 % (i.e. 35–43 mg/kg). The previous recommendation for the amount of iodine to add to salt was 20–40 mg/kg using an estimated salt intake of 10 g/d^(^ ^15^ ^)^.

As noted above, seventeen countries have passed regulations under their existing Food Act/Law. As such, standards or technical regulations for salt, such as iodine, moisture or purity levels, are specified in the existing salt iodization legislation. Eleven countries have ‘stand-alone’ legislation or regulations under an existing alternative law. Of these countries, four (Afghanistan, Bangladesh, Cambodia and Timor Leste) have included salt iodization standards in the ‘stand-alone’ law. Other countries have included iodization standards in a separate standard for salt. This has its advantages as it will likely be easier to change the salt/iodization standard than changing the original law.

**Fig. 1:**
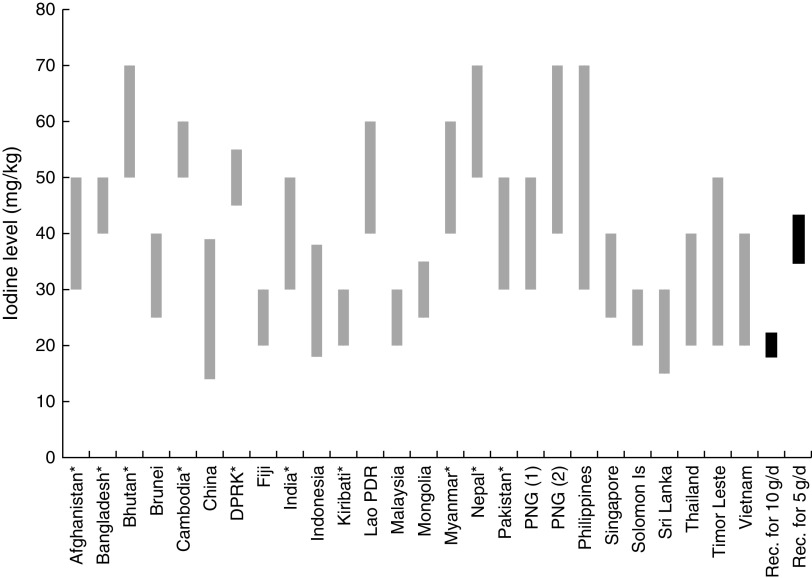
Required levels of iodine in salt, based on national standards (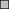), and WHO recommended (Rec.) iodization levels depending on salt intake (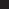). *Production/import level specified. Bhutan, India, Indonesia and Nepal have not specified an upper limit; for illustrative purposes, an upper limit of 20 ppm above the lower limit has been used. PNG has standards for two categories of salt: (1)=salt; (2)=table salt. China national standard shown; provinces choose provincial standards within this range (DPRK, Democratic People’s Republic of Korea; Lao PDR, Lao People’s Democratic Republic; PNG, Papua New Guinea)

Required levels for iodization at production/import level are shown in Fig. 1. It was found that while all countries have standards for iodization, not all countries specified the level at which the iodization standard should be applied – production/import, retail or household. Figure 1 also illustrates WHO recommended levels for iodization assuming salt intake from table salt and processed foods is 10 g/capita per d or 5 g/capita per d. As Fig. 1 shows, most counties in Asia have significantly higher salt iodization standards than currently recommended by WHO, particularly if salt consumption is closer to 10 g/capita per d.

### Other salt standards

The legislation reviewed often also included standards for aspects of salt other than the iodine level, such as maximum levels of insoluble material, contaminants such as calcium or magnesium chloride and calcium sulfate, or heavy metals such as arsenic and mercury. Most standards also specified sodium chloride (NaCl) content and allowable moisture levels. Studies on the stability of iodine in iodized salt have indicated that iodine is lost more rapidly from salt with a high moisture content and from salt with impurities such as magnesium. Iodine retention is better in higher-quality salt^(^ ^20^ ^)^.

### Fortificant

The majority of legislation stipulates what iodine compound should be used for salt iodization, either in the original legislation or, more usually, in the salt standard. Most have stipulated potassium iodate but many also allow potassium iodide, sodium iodate and/or sodium iodide. Very seldom does the legislation specify who is responsible for procuring the fortificant. In practice, in many countries either a donor or the government has taken on responsibility for the cost of the fortificant, particularly at the start of the programme. The Ministry of Finance pays for potassium iodate in China, it was provided free by the Hospital of Endocrinology in Vietnam until 2005, and the Food and Drug Administration in Thailand is currently providing it for free on a temporary basis. UNICEF or other donors have provided it for free in numerous countries including Cambodia, DPRK, Lao PDR, Mongolia and Myanmar. Difficulties have been experienced in several countries in ending free or subsidized provision of potassium iodate and in a number of countries, such as Cambodia^(^ ^21^ ^)^ and Vietnam^(^ ^22^ ^)^, coverage has declined following the withdrawal of donations of potassium iodate.

### Coordination and implementation stipulations

Salt iodization requirements legislated through food regulations under Food Acts/Laws indicate standards for salt, including iodization, but implementation details are not included, as routine food control systems apply. Conversely, stand-alone legislation and supporting regulations include varying detail on how salt iodization should be implemented. Implementation details include programme oversight, requirements for salt producers, enforcement responsibilities, regulation on import or transportation of salt, responsibilities for public education and penalties.

### Salt iodization achievements in the region

Compared with other regions in the world, the East Asia and Pacific and South Asia regions have the highest proportion of households using iodized salt: 86 and 69 %, respectively^(^ ^5^ ^)^.

The situation in Malaysia, Vietnam and India clearly supports global evidence that mandatory legislation is more effective. Salt iodization is mandatory in only two of Malaysia’s sixteen states where IDD was thought to be endemic. A national survey in 2008 found that while 82 % of households consumed iodized salt in the areas covered by mandatory legislation, only 21 % consumed iodized salt in the rest of the country with voluntary legislation^(^ ^23^ ^)^. In Vietnam, salt iodization was mandatory until 2005, at which time 93 % of households were using adequately iodized salt. Under the subsequent period of voluntary iodization (2005–2016), however, the proportion of households using iodized salt rapidly declined to 45 % in 2010^(^ ^22^ ^)^. In India, a federal ban on the sale of common (non-iodized) salt existed under the Food Adulteration Act from 1998 to 2000. In 2000, however, the Food Adulteration Act was repealed and between 2000 and 2006, India had no legislation on salt iodization until the Food Standards and Safety Act of 2006 reinstated the ban on non-iodized salt. A survey in 1998/99, when non-iodized salt was banned under the Food Adulteration Act, found household use of iodized salt to be 49 %. It fell to 30 % in 2002–2004 when there was no ban and rose to 51, 71 and 78 %, respectively, in 2005/06, 2009 and 2014/15 after the Food Standards and Safety Act came into force^(^ ^24^ ^)^.*

**Fig. 2:**
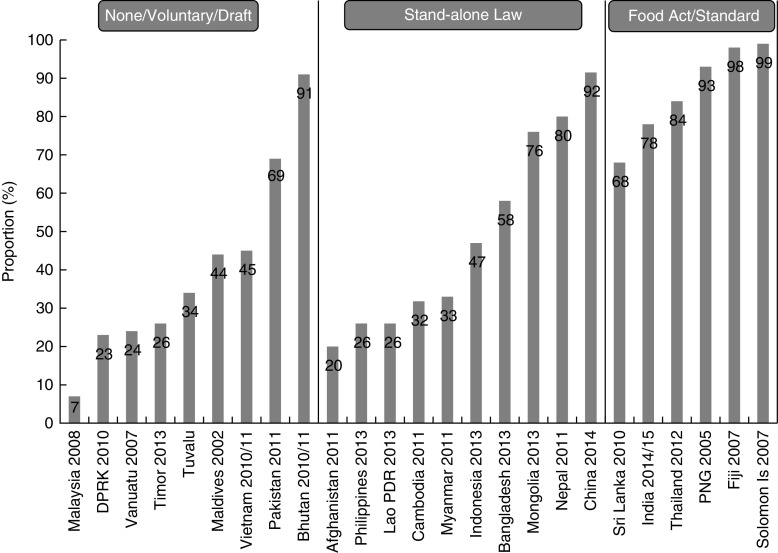
Proportion of households consuming adequately iodized salt by type of salt iodization legislation. Note: Household availability of adequately iodized salt assessed by quantitative method against national standard. Exceptions are Fiji, Tuvalu and Vietnam which used semi-quantitative rapid test kits. Source of data is most recent national survey (date of survey indicated) with quantitative assessment of iodine content, except Solomon Islands which is non-national (Honiara only) and Thailand which is surveillance data. Malaysia has been included in the None/Voluntary/Draft category as it does not currently have national legislation (DPRK, Democratic People’s Republic of Korea; Lao PDR, Lao People’s Democratic Republic; PNG, Papua New Guinea)

Figure 2 shows most recent data on household consumption of adequately iodized salt by country, grouped according to the type of legislation the countries have. Countries with no, draft or voluntary legislation clearly have lower coverage than those with regulations under the Food Act/Law. Relatively high coverage in Bhutan and Pakistan may be attributable to the national policy which bans the importation of non-iodized salt into Bhutan and the existence of mandatory legislation in some provinces of Pakistan. While several countries with stand-alone legislation have high coverage, particularly China, on average, coverage in countries with stand-alone legislation is lower than in those with regulations under the Food Act/Law. Information from the countries with lowest coverage in the stand-alone category indicates problems with enforcement. China and Nepal have rather unique circumstances: the Chinese salt industry was highly regulated by the government until the start of 2017, which has contributed significantly to the success of the salt iodization programme^(^ ^25^ ^)^. Nepal imports all of its salt and iodizes any non-iodized or inadequately iodized salt in a limited number of government-managed iodization factories at the border.

## Discussion

The current review found that most countries in the Asia and Pacific regions have mandatory salt iodization legislation. Several others are planning mandatory legislation and some have updated and strengthened existing legislation. Countries have chosen to have stand-alone laws that include extensive implementation details, or have simply incorporated the requirement for iodization into national salt regulations. Data on household coverage with adequately iodized salt in the region suggest that it is higher in countries with salt iodization regulations under the Food Act/Law. It can be argued that stand-alone legislation has the propensity to establish salt iodization as a vertical intervention, with potential constraints on implementation, while requiring fortification through food standards leads to implementation through the routine food control system^(^ ^25^ ^)^. Some authors have argued that regulatory monitoring systems for fortified foods are often not effective^(^ ^26^ ^)^ but establishing effective monitoring systems within the routine food control system is likely easier than establishing completely new and separate systems, which may occur with stand-alone legislation.

Evidence is clear that mandatory food fortification legislation is more effective than voluntary legislation^(^ ^11^ ^)^. Our review suggests that in Asian countries, legislating fortification through food regulations under the Food Act/Law facilitates implementation, in particular external regulatory monitoring and enforcement. It is also clear that legislation alone cannot assure a successful programme. Mandatory legislation for rice fortification has also not assured successful implementation^(^ ^27^ ^)^. Legislation is effective only if consistently and fairly enforced. For this to happen, political commitment, effective coordination systems, effective monitoring and enforcement systems, and a conducive salt industry are needed.

In Asia, legislation that bans non-iodized food-grade salt or requires all food-grade salt to be iodized is more effective than legislation which tries to legislate how all or some salt should be iodized. In particular, it is suggested that setting conditions for salt iodization hinders iodization of all salt. For example, requiring salt producers to register or have a licence can be counterproductive as such requirements provide a loophole for all those who choose not to register or do not qualify for a licence. If governments choose to try to control the industry in this way, the political commitment and enforcement capacity must exist to close all facilities not registered or licensed; there is no evidence in any of the countries reviewed that this is happening. Similarly, several Asian countries have established standards for salt that appear to be higher than the domestic salt industry can achieve, particularly in relation to NaCl and heavy metal content. Enforcement of such standards would drive many salt producers out of business and reduce the availability of domestic salt. In reality, it appears that these standards are not being enforced, which again creates a loophole for salt processors; if they are not required to meet standards for say NaCl or moisture content, why would they be expected to meet standards for iodization?

Changes in eating patterns and salt consumption highlight the importance of iodizing the salt used in food processing, in addition to table salt. Salt used in food processing may be particularly important in the East Asia region where use of salty condiments such as soya sauce, fish sauce, fermented fish, soyabean paste, etc., instead of table salt, is widespread. Limited data from Vietnam found that 35 % of total sodium intake was from seasonings, 32 % was from fish sauce and 8 % was from instant noodles, with only 6 % from table salt^(^ ^22^ ^)^. It is worrying therefore that some countries exclude salt for food processing, while it is ambiguous or not specified in others.

Comparison of salt iodization standards in the region with WHO recommended levels suggests that iodization standards should be lowered in several countries. It is likely that standards have been set high to compensate for losses of iodine from low-quality salt. However, WHO guidelines have assumed 30 % loss of iodine between production and consumption^(^ ^8^ ^)^. It is likely that iodine losses in the region are considerably less than assumed, especially if the salt is appropriately packaged. Countries should use median urinary iodine data to assess whether salt iodization levels are appropriate, including looking at sub-population groups consuming salt with different levels of iodine. Such data on iodine status should be used to evaluate the impact of salt iodization programmes, to ensure all population groups have adequate iodine status and none have excess.

## Conclusions

In line with global data which indicate that the majority of countries have mandatory legislation for at least some types of salt^(^ ^28^ ^)^, the current review found the majority of countries in the Asia region to have mandatory salt iodization legislation. Our review highlights the importance of having legislation to achieve higher coverage at household level. Legislation under national food laws of the country appears to have facilitated higher coverage of adequately iodized salt in Asia, apparently because it has helped to establish requirements for iodization and systems of enforcement into the routine food control system. On the other hand, stand-alone legislation tends to establish salt iodization as a vertical programme and implementation modalities of some programmes in the region are hindering enforcement and sustainability. Standards for salt and salt iodization appear to be higher than the global norm or recommendations.

Adequate and appropriate salt iodization legislation can facilitate a successful intervention, but the impact will be limited if legislation is not adequately enforced. Additional facilitating factors are needed including adequate political commitment, necessary capacity and support to the salt industry, and monitoring and evaluation systems that identify constraints and problems and guide programme strengthening.

## Footnotes

* That is, countries in UNICEF East Asia and Pacific and South Asia regions.

† Personal communication in April 2015 with Peter Hoejskovp, Technical Officer – Food Safety, Division of Health Security and Emergencies, WHO Regional Office for the Western Pacific, Manila, Philippines. Copies of the draft legislation were not made available.

‡ Khyber Pakhtunkhwa and Baluchistan provinces have amended the Pure Food Act to ban non-iodized food-grade salt; Gilgit-Baltistan has passed an IDD Control Act (2011) creating a IDD Control Committee to oversee a ban on non-iodized food-grade salt; Sindh has issued the Sindh Compulsory Iodization of Salt Act; and Azad Jammu and Kashmir have issued a Public Health Secretariat Notification banning the sale of non-iodized salt.

§ Personal communication in March 2017 with Zalma Bt Abdul Razak, Director, Nutrition Division, Ministry of Health Malaysia.

†† Singapore is not considered the first country as it does not have USI legislation; it has a food standard that allows voluntary fortification of salt with iodine.

‖ Ministry of Health (2009) Draft Operational guideline on sustaining iodine deficiency disorders elimination.

¶ State Planning Committee of the Democratic People’s Republic of Korea (2015) Plan for Universal Salt Iodization in DPRK 2016–2021 (draft).

** Ministry of Health and Gender, Micronutrient Policy and Strategy (2012) Food regulations and standards 2011 (draft).

* Codling K for UNICEF and GAIN India (2013) Salt Iodization in India: An Analysis of the Situation in Uttar Pradesh and Tamil Nadu. UNICEF India (unpublished report).

## References

[1] United Nations System Standing Committee on Nutrition (2007) Universal Salt Iodization: Global progress and public health success stories to address IDD through USI, key programme components, lessons learned at country level, and the way forward to reach USI globally. *SCN News* no. 35, end 2007. http://189.28.128.100/dab/docs/portaldab/documentos/scnnews35.pdf (accessed May 2017).

[2] Iodine Global Network (2016) Global Scorecard for Iodine Nutrition in 2016. http://www.ign.org/cm_data/Scorecard_2016_SAC_PW.pdf (accessed May 2017).

[3] UNICEF & World Health Organization (1994) World Summit for Children – Mid-Decade Goal: Iodine Deficiency Disorders. Report of UNICEF–WHO Joint Committee on Health Policy, Special Session, Geneva, January 1994. Geneva: UNICEF/WHO; available at http://www.ceecis.org/iodine/01_global/01_pl/01_01_1994_summit.pdf

[4] UNICEF (2001) Progress Since the World Summit for Children: A Statistical Review. New York: UNICEF; available at http://www.unicef.org/publications/index_6440.html

[5] UNICEF (2016) The State of the World’s Children 2016: A Fair Chance for Every Child. New York: UNICEF; available at https://www.unicef.org/publications/index_91711.html

[6] Horton S, Alderman H & Rivera J (2008) Copenhagen Consensus 2008 Challenge Paper: Hunger and Malnutrition. http://www.copenhagenconsensus.com/sites/default/files/CP_Malnutrition_and_Hunger_-_Horton.pdf (accessed May 2017).

[7] Aburto N, Abudou M, Candeias V et al. (2014) Effect and Safety of Salt Iodization to Prevent Iodine Deficiency Disorders: A Systematic Review with Meta-Analyses. Geneva: WHO; available at http://apps.who.int/iris/bitstream/10665/148175/1/9789241508285_eng.pdf

[8] World Health Organization (2014) Guideline: Fortification of Food-Grade Salt with Iodine for the Prevention and Control of Iodine Deficiency Disorders. Geneva: WHO; available at http://www.who.int/nutrition/publications/guidelines/fortification_foodgrade_saltwithiodine/en/ 25473709

[9] World Health Organization (2013) Salt Reduction and Iodine Fortification Strategies in Public Health: Report of a Joint Technical Meeting Convened by the World Health Organization and The George Institute for Global Health in Collaboration with the International Council for the Control of Iodine Deficiency Disorders Global Network, Sydney, Australia, March 2013. Geneva: WHO; available at http://apps.who.int/iris/bitstream/10665/101509/1/9789241506694_eng.pdf

[10] Gautam K (2007) Global progress in addressing iodine deficiency through USI: the makings of a global public health success story – the second decade (1995–2007). In Universal Salt Iodization: Global progress and public health success stories to address IDD through USI, key programme components, lessons learned at country level, and the way forward to reach USI globally. *SCN News* no. 35, end 2007, pp. 12–18. http://189.28.128.100/dab/docs/portaldab/documentos/scnnews35.pdf (accessed May 2017).

[11] Hoogendoom A, Luthringer C, Parvanta I et al. (2017) Food Fortification: Global Mapping Study 2016. https://ec.europa.eu/europeaid/food-fortification-global-mapping-study-2016_en (accessed May 2017).

[12] UNICEF (2008) Sustainable Elimination of Iodine Deficiency. New York: UNICEF; available at https://www.unicef.org/publications/index_44271.html

[13] Vietnam News Agency (2016) Compulsory micronutrients on food introduced. http://vietnam.vnanet.vn/english/compulsory-micronutrients-on-food-introduced/220445.html (accessed May 2017).

[14] UNICEF (1990) Major Goals for Child Survival, Development and Protection. http://www.unicef.org/wsc/goals.htm#Nutrition (accessed May 2017).

[15] World Health Organization, UNICEF & International Council for Control of Iodine Deficiency Disorders (2007) Assessment of Iodine Deficiency Disorders and Monitoring their Elimination: A Guide for Programme Managers, 3rd ed. Geneva: WHO; available at http://apps.who.int/iris/bitstream/10665/43781/1/9789241595827_eng.pdf

[16] National Center for Chronic Disease Prevention and Health Promotion, Division for Heart Disease and Stroke Prevention, Centers for Disease Control and Prevention (2016) Get The Facts: Sodium’s Role in Processed Food. http://www.cdc.gov/salt/pdfs/sodium_role_processed.pdf (accessed May 2017).

[17] World Iodine Association (2017) Animal Nutrition. http://www.worldiodineassociation.com/animals/ (accessed May 2017).

[18] Federal Commission for Nutrition (2013) Iodine Supply in Switzerland: Current Status and Recommendations. Expert Report of the FCN. Zurich: Federal Office of Public Health; available at https://www.eek.admin.ch/dam/.../expertenbericht.../EEK_Iodbericht_final_9-5.pdf

[19] World Health Organization (2012) Guideline: Sodium Intake for Adults and Children. Geneva: WHO; available at http://www.who.int/nutrition/publications/guidelines/sodium_intake_printversion.pdf 23658998

[20] Diosady LL, Alberti JO, Venkatesh Mannar MG et al. (1998) Stability of iodine in iodized salt used for correction of iodine-deficiency disorders. Food Nutr Bull 19, 240–250.

[21] Arnaud L, Mam B, Oeurn S et al. (2015) Iodized salt in Cambodia: trends from 2008 to 2014. Nutrients 7, 4189–4198. 26035245 10.3390/nu7064189 PMC4488780

[22] Codling K, Quang NV, Phong L et al. (2015) The rise and fall of universal salt iodization in Vietnam: lessons learned for designing sustainable food fortification programmes with a public health impact. Food Nutr Bull 36, 441–454. 26578534 10.1177/0379572115616039

[23] Selamat R, Mohamud WN, Zainuddin AA et al. (2010) Iodine deficiency status and iodized salt consumption in Malaysia: findings from a national iodine deficiency disorders survey. Asia Pac J Clin Nutr 19, 578–585. 21147721

[24] Pandav C, Yadav K, Lakshmy R et al. (2015) Across India, women are iodine sufficient. Excerpt from National Iodine and Salt Intake Survey (NISI) 2014–2015: Executive Summary. http://www.ign.org/cm_data/IDD_nov15_india.pdf (accessed July 2017).

[25] Sun D, Codling K, Chang S et al. (2017) Eliminating iodine deficiency in China: achievements, challenges and global implications. Nutrients 9, E361. 10.3390/nu9040361 PMC5409700 28379180

[26] Makhumula P, Dary O, Guamuch M et al. (2014) Legislative frameworks for corn flour and maize meal fortification. Ann N Y Acad Sci 1312, 91–104. 24521440 10.1111/nyas.12349

[27] Luthringer CL, Rowe LA, Vossenaar M et al. (2015) Regulatory monitoring of fortified foods: identifying barriers and good practices. Glob Health Sci Pract 3, 446–461. 26374804 10.9745/GHSP-D-15-00171 PMC4570017

[28] Food Fortification Initiative, Global Alliance for Improved Nutrition & Iodine Global Network (2017) Global Repository on Food Fortification, Version 1. www.fortificationdata.org

